# Cholesterol efflux capacity is associated with lipoprotein size and vascular health in mild to moderate psoriasis

**DOI:** 10.3389/fcvm.2023.1041457

**Published:** 2023-02-20

**Authors:** Alexander R. Berg, Rylee F. Petrole, Haiou Li, Alexander V. Sorokin, Alvaro Gonzalez-Cantero, Martin P. Playford, Nehal N. Mehta, Heather L. Teague

**Affiliations:** ^1^National Heart, Lung, and Blood Institute, NIH, Bethesda, MD, United States; ^2^Dermatology Service, Hospital Universitario Ramón y Cajal, Medicine Department, Faculty of Medicine, Universidad de Alcalá, IRYCIS, Madrid, Spain; ^3^Faculty of Medicine, Universidad Francisco de Vitoria, Pozuelo de Alarcón, Madrid, Spain

**Keywords:** psoriasis, cholesterol efflux, high density lipoprotein, low density lipoprotein, lipoprotein size, atheroscelrosis

## Abstract

**Background and objective:**

Psoriasis is a systemic inflammatory condition with poor cholesterol transport measured by cholesterol efflux capacity (CEC) that is associated with a heightened risk of cardiovascular disease (CVD). In psoriasis patients, we sought to characterize the lipoprotein profile by size using a novel nuclear magnetic resonance algorithm in patients with low CEC compared to normal CEC.

**Methods:**

Lipoprotein profile was assessed using the novel nuclear magnetic resonance LipoProfile-4 deconvolution algorithm. Aortic vascular inflammation (VI) and non-calcified burden (NCB) were characterized *via* positron emission tomography-computed tomography and coronary computed tomography angiography. To understand the relationship between lipoprotein size and markers of subclinical atherosclerosis, linear regression models controlling for confounders were constructed.

**Results:**

Psoriasis patients with low CEC had higher more severe psoriasis (*p* = 0.04), VI (*p* = 0.04) and NCB (*p* = 0.001), concomitant with smaller high-density lipoprotein (HDL) (*p* < 0.001) and low-density lipoprotein (LDL) particles (*p* < 0.001). In adjusted models HDL size (*β* = −0.19; *p* = 0.02) and LDL size (*β* = −0.31; *p* < 0.001) associated with VI and NCB. Lastly, HDL size strongly associated with LDL size in fully adjusted models (*β* = −0.27; *p* < 0.001).

**Conclusion:**

These findings demonstrate that in psoriasis, low CEC associates with a lipoprotein profile comprised of smaller HDL and LDL particles which correlates with vascular health and may be driving early onset atherogenesis. Further, these results demonstrate a relationship between HDL and LDL size and provide novel insights into the complexities of HDL and LDL as biomarkers of vascular health.

## Introduction

In 1987, the Helsinki Heart Study reinvigorated interest in high-density lipoprotein (HDL) by evaluating the efficacy of increasing HDL while simultaneously decreasing low-density lipoprotein (LDL) in reducing the risk of coronary artery disease (1, 2). Positive outcomes prompted elaborate studies of HDL to further understand how knowledge about HDL can be implemented in a clinical setting (3), however, pharmacologic increases in HDL levels have not proven beneficial (4, 5). Conversely, the growing body of literature has strongly supported lipid-lowering therapies targeting LDL to reduce cardiac event rates (6). Nonetheless, HDL and LDL remain independent risk factors for coronary artery disease and their interaction has proven a more accurate predictor (7, 8).

In-depth investigations have revealed the complexities of HDL, suggesting current study designs emphasize HDL function and size for the development of therapeutic strategies to improve cardiovascular (CV) outcomes (9). HDL is a heterogenous population of particles containing equal amounts of lipid and protein that vary in size from 7.5 to 13 nanometers (nm) (3, 10). To date, upwards of 500 proteins have been identified to be associated with HDL, depending upon the size and concentration (9, 11). The structure and composition of LDL is much less complex, containing mostly apolipoprotein B100 (ApoB100), phospholipids, cholesterol, and triglycerides (12). Functionally, HDL and LDL operate as part of a biological system responsible for maintaining cholesterol homeostasis throughout the tissues by delivering cholesterol to the tissues and returning it to the liver for bile acid synthesis and excretion (3). In brief, HDL removes excess cholesterol form peripheral tissues, passes cholesterol to LDL through cholesteryl ester transfer protein (CETP) and LDL delivers cholesterol to the liver. Disruptions in this biological framework leads to lipid deposition in the vessel wall concomitant with an upregulation of myelopoiesis in the bone marrow, driving systemic inflammation (13). Restoration of HDL function in animal models restores cholesterol homeostasis and thereby reduces inflammation and atherosclerotic plaque formation (13, 14).

Systemic inflammation can have detrimental effects on HDL function (9). The inflammatory microenvironment causes dissociation of Apo-A1 from HDL (15), leads to chemical modifications of Apo-A1, and thereby mitigates HDLs functional capabilities (16). Further, smaller HDL and LDL particles, depicted as more atherogenic, tend to concentrate in inflammatory environments (17, 18). Psoriasis is a systemically inflamed population characterized by impairments in HDL cholesterol efflux capacity (CEC) that directly correlates with vascular health (18, 19). Additionally, in psoriasis, bile acid metabolism is reduced (20) and oxidized LDL accumulates in the skin (21). The interaction between HDL and LDL is undoubtedly implicated, however, comprehensive analyses of HDL function, lipoprotein size and the relationship with vascular health in psoriasis are incomplete. Recent advances in nuclear magnetic resonance (NMR) technology allows for the computation of a comprehensive lipid panel encompassing precise quantifications of lipoproteins and resolution of their subgroups according to size (22). Thus, important relationships between lipoprotein function, size and vascular health in an inflammatory microenvironment can be identified (9).

Patients with psoriasis have accelerated cardiovascular (CV) risk not captured by traditional risk factors and experience myocardial infarctions at a younger age (23, 24). The severity of psoriasis is relative to their vascular health characterized by fluorodeoxyglucose (FDG)-positron emission tomography (PET) uptake in the aorta and non-calcified burden (NCB) in the coronary arteries by coronary computed tomography angiography (CCTA) (25, 26). While vascular FDG-uptake in sub-clinical atherosclerosis occurs at sites of inflammation, aortic FDG-uptake correlates with NCB detected by CCTA, a non-invasive, high-quality assessment of subclinical atherosclerosis (27, 28).

Given the previously reported relationships between HDL function and vascular disease in psoriasis, we sought to understand the comprehensive lipoprotein profile in patients with poor HDL function and more prevalent vascular inflammation and coronary burden. To our knowledge, this is the first clinical investigation assessing the relationships between vascular disease and both lipoprotein function and size in psoriasis.

## Materials and methods

### Study approval and design

Our study enrolled 310 consecutive participants who were recruited as part of the Psoriasis Atherosclerosis and Cardiometabolic Disease Initiative (PACI) from January 2013 to February 2022 (NCT01778569). The detailed inclusion and exclusion criteria of the study have been previously reported (29). All study protocols were approved by the institutional review boards (IRB) of the National Heart, Lung, and Blood Institute and completed in accordance with the Declaration of Helsinki Principles. Written informed consent was received from participants prior to inclusion in the study. Biologic therapies for psoriasis treatment included the following agents: TNF-α inhibitors (adalimumab, etanercept), interleukin IL-12/23 inhibitor (ustekinumab), and IL-17 inhibitors (ixekizumab, secukinumab). There were no changes in biologic status or type of biologic therapy throughout the duration of the study. The study and study results were reported in accordance with the Strengthening the Reporting of Observational Studies in Epidemiology (STROBE) (30).

### Clinical measurements

Each psoriasis patient underwent fasting blood draws for CEC measurements, inflammatory markers and cytokines, and lipid profiling, the same day as the CCTA and FDG/PET scans. Psoriasis severity was assessed by a dermatologist to determine PASI. Laboratory parameters including fasting blood glucose, fasting lipid panel, white blood count with differential, and systemic inflammatory markers including Hs-CRP were evaluated in a clinical laboratory (31). Glycoprotein acetylation (GlycA) was measured *via* a Vantera clinical nuclear magnetic resonance analyzer using the LipoProfile-3 algorithm (Labcorp) (32). Plasma cytokines were measured in EDTA-plasma utilizing the MesoScale Discovery platform (MSD, Gaithersburg, MD).

### Aorta and bone marrow measured by 18F- 18F-FDG-Pet/CT

18F-FDG-PET/CT imaging was performed using one Siemens Biograph mCT PET/CT 64-slice scanner (Siemens Medical Solutions USA, Malvern, Pennsylvania) at a single center. After an overnight fast for a minimum of 8 h, PET/CT images were acquired approximately 60 min (mean: 62 ± 1 min) after administration of 18F-FDG. All patients underwent the same PET/CT protocol with the same team of technologists with a fixed 18F-FDG dose of 10 mCi. Standard bed positions of 3 min each, scanning cranially to caudally, were obtained for each patient. 1.5-mm axial slices were obtained. Patients with a fasting glucose over 200 mg/dL were excluded.

Aorta and bone marrow 18F-FDG uptake were quantified using previously published methods using a dedicated PET/CT image analysis program [OsirixTM version 5.8.5 (Pixmeo SARL, Geneva, Switzerland)] (33). The average standardized uptake value (SUVmax) of the 17 individual vertebrae was recorded. The SUVmax was generated using a dedicated PET/CT image analysis program (OsirixTM version 5.8.5 (Pixmeo SARL, Geneva, Switzerland)). Intra-reader and inter-reader variability and bias were minimal.

### Coronary plaque parameters measured by coronary computed tomography angiography

To assess NCB, participants underwent CCTA using the same scanner (320-detector row Aquilion ONE ViSION, Toshiba, Japan). NCB was assessed separately in all three main coronary arteries greater than 2 mm diameter (left anterior descending, left circumflex, and right coronary artery) using QAngio CT (Medis, Netherlands). Total burden (TB) and NCB indices were calculated by dividing total vessel plaque volume by total vessel length to account for variable coronary artery lengths and subsequently adjusted for mean luminal 10 intensity to yield TB and NCB using adaptive threshold for cut-off values (34). Guidelines implemented by the NIH Radiation Exposure Committee were adhered to.

### High-density lipoprotein cholesterol efflux capacity

High-density lipoprotein cholesterol efflux capacity assessment was based on published methods (35). In brief, J774 cells were radiolabeled with 2 μCi of 3H-cholesterol/mL. ATP-binding cassette transporter A1 (ABCA1) levels were upregulated through a 16-h incubation with 0.3 mmol/L 8-(4-chlorophenylthio)-cAMP. Then, 2.8% apoB-depleted plasma, depleted by polyethylene glycol 6,000, was added to the efflux medium for 4 h. To quantify the efflux of radioactive cholesterol from the cells, liquid scintillation counting was used. CEC was calculated by using the following formula: (μCi of 3H-cholesterol in media containing 2.8% apoB-depleted subject plasma – μCi of 3H-cholesterol in plasma-free media/μCi of 3H-cholesterol in media containing 2.8% apoB-depleted pooled control plasma – μCi of 3H-cholesterol in pooled control plasma-free media). Samples were assayed in duplicate.

### Advanced LipoProfile 4 algorithm

The lipoprotein profile was measured on a plasma sample from a subgroup of psoriasis patients (*n* = 213) collected at baseline. NMR LipoProfile^®^ analysis was preformed using the LP4 deconvolution algorithm on the Vantera^®^ NMR Clinical Analyzer (LabCorp^®^, Burlington, NC, United States) (36). The advanced LP4 algorithm was utilized to quantitate four types of measurements; (1) the overall particle size for HDL and LDL; (2) the total concentration of each lipoprotein group (HDL, 7.5–13 nm, LDL, 19–23 nm); (3) the concentration of each lipoprotein subgroup (large, medium, and small); (4) the concentration of subgroups broken down into smaller fractions.

### Statistical analysis

For this study, values are reported as mean (standard deviation) for parametric variables, median (interquartile range) for non-parametric variables, and *n* (%) for categorical variables. Statistical significance was assessed by a student’s *t*-test or a Wilcoxon rank-sum test and corresponding Kruskal-Wallis’s test for nonparametric variables. Pearson’s *χ*^2^ test was used for categorical variables. Spearman correlation coefficient was applied to measure the association between variables of interests. To elucidate the relationship between HDL or LDL size and CEC, VI, and NCB, simple and multivariate linear regression analysis were conducted by including possible cofounders. NCB was log-transformed to meet the normality assumption. Models were fully adjusted for age, sex, waist to hip ratio, CEC, hs-CRP, statin treatment and biologic therapy. Analyses were performed with StataIC 12 (Stata Corp., College Station, TX, United States).

## Results

### In psoriasis, patients with low cholesterol efflux capacity have an altered metabolic, inflammatory, and cardiovascular disease profile

A cohort of 310 patients with psoriasis was stratified by median HDL CEC (>0.95, high CEC, ≤0.95 low CEC) and their metabolic, inflammatory, and cardiovascular parameters were compared (Table 1). The low CEC and high CEC groups were similar in age with a higher prevalence of males (*p* = 0.002). The low CEC group had an increased prevalence of metabolic syndrome, a higher BMI, a larger waist-to-hip ratio, and more severe psoriasis (all *p* < 0.05) compared to the high CEC group. In the low CEC group, insulin signaling was disrupted, with increased glucose (*p* = 0.02) elevated insulin levels and a higher homeostatic model for insulin resistance (HOMA-IR; both *p* < 0.001) suggesting insulin resistance in the poor CEC group. Compared to the high CEC group, the low CEC group had less total, HDL and LDL cholesterol (*p* ≤ 0.01).

**Table 1:** Clinical characteristics of psoriasis patients stratified by median CEC (0.95).

Demographics and clinical characteristics	Total (*n* = 310)	Low CEC (*n* = 155)	High CEC (*n* = 155)	*p*-value
Age, years	49.5 ± 12.9	49.0 ± 13.1	50.0 ± 12.7	0.50
Males, *n* (%)	185 (60)	106 (68.4)	79 (51)	0.002**
Current smoker, *n* (%)	38 (12)	21 (13.5)	17 (11.0)	0.49
Hypertension, *n* (%)	82 (27)	42 (27.1)	40 (25.8)	0.80
Hyperlipidemia, *n* (%)	113 (37)	56 (36.1)	57 (36.8)	0.91
Type-2 Diabetes, *n* (%)	38 (12)	22 (14.2)	16 (10.3)	0.30
Metabolic Syndrome, *n* (%)	92 (31)	57 (37.5)	35 (23.5)	0.008**
FRS	2.0 (0.5–5.6)	2.4 (0.5–7.4)	1.7 (0.5–4.8)	0.08
Statin Use, *n* (%)	75 (24)	41 (26.5)	34 (21.9)	0.35
BMI (kg/m^2^)	28.4 (24.9–32.6)	29.3 (26.1–34.5)	27.2 (24–30.3)	<0.001***
Waist-to-hip ratio	1.0 (0.9–1.0)	1.0 (0.9–1.0)	0.9 (0.9–1.0)	<0.001***
SBP, mm Hg	122 (112–129)	122 (112–130)	122 (112–129)	0.93
DBP, mm Hg	72 (66–78)	73 (65–78)	71 (66–77)	0.59
**Psoriasis characterization**				
PASI score	6.0 (3.1–10.6)	6.6 (3.4–12.6)	5.4 (2.8–9.6)	0.04*
Systemic treatment, *n* (%)	43 (14)	17 (11.0)	26 (16.8)	0.14
Biologic treatment, *n* (%)	99 (32)	45 (29.0)	54 (34.8)	0.27
**Metabolic and lipoprotein characteristics**				
Glucose	96 (90–105)	98 (92–107)	94 (88–105)	0.02*
Insulin	10.8 (6.8–17.3)	12.6 (7.8–20.6)	9.9 (6.2–14.9)	<0.001***
HOMA-IR	2.7 (1.6–4.5)	3.2 (1.9–5.2)	2.2 (1.4–3.8)	<0.001***
CEC	0.96 (0.87–1.09)	0.9 (0.8–0.9)	1.1 (1.0–1.2)	<0.001***
Total cholesterol, mg/dL	183 (159–209)	171 (152–192)	195 (168–223)	<0.001***
HDL cholesterol, mg/dL	52 (44–64)	47 (40–56)	60 (50–75)	<0.001***
LDL cholesterol, mg/dL	105 (83–123)	98 (80–116)	110 (86–129)	0.01*
**Inflammatory Markers and Cytokines**				
C-reactive protein, mg/L	1.8 (0.8–4.1)	2.2 (0.9–4.8)	1.5 (0.7–3.3)	0.04*
GlycA	399 (355–450)	399 (359–450)	398 (350–449)	0.51
Absolute Neutrophils, K/uL	3.65 (2.90–4.53)	3.79 (3.12–4.83)	3.52 (2.62–4.30)	0.01*
IL6, pg./mL	1.4 (0.8–2.3)	1.6 (0.9–2.9)	1.3 (0.8–2.1)	0.05*
TNF-α, pg./mL	1.5 (0.9–2.9)	1.6 (1.1–3.6)	1.4 (0.8–2.3)	0.04*
**PET-CT Parameters**				
Aortic Vascular (TBR)	1.63 (1.51–1.80)	1.70 (1.52–1.85)	1.60 (1.51–1.76)	0.04*
Bone Marrow (SUV_max_)	4.23 (3.59–5.32)	4.36 (3.75–5.56)	4.15 (3.27–5.09)	0.05*
**Vascular Characterization**				
TB, mm^2^ (×100)	1.10 (0.88–1.46)	1.22 (0.95–1.58)	1.02 (0.85–1.30)	0.001*
NCB, mm^2^ (×100)	1.05 (0.84–1.42)	1.12 (0.87–1.52)	0.95 (0.81–1.26)	0.001*
DCB, mm^2^ (×100)	0.02 (0.01–0.05)	0.02 (0.01–0.05)	0.02 (0.01–0.05)	0.77

Values reported in the table as means ± SD or median (IQR) for continuous variables and as *n* (%) for categorical variables. Low CEC defined as ≤ 0.95 (median CEC). *p ≤ 0.05, **p < 0.01, ***p < 0.001 deemed significant. CEC, cholesterol efflux capacity; FRS, 10-year Framingham risk score; BMI, body mass index; SBP, systolic blood pressure; DBP, diastolic blood pressure; PASI, psoriasis area severity index; HOMA-IR, homeostatic model for insulin resistance; HDL, high density lipoprotein; LDL, low density lipoprotein; GlycA, glycoprotein A; K, 1000; IL-6, interleukin-6; TNF-α, tumor necrosis factor alpha; TB, total burden; NCB, non-calcified coronary burden; DCB, dense calcified burden.

Analyses of the inflammatory profile revealed the low CEC group had higher C-reactive protein, more circulating neutrophils and higher levels of interleukin-6 (IL6) and tumor necrosis factor-alpha (TNF-α; *p* < 0.05). FDG-uptake was accelerated in the aorta and bone marrow of patients with low CEC compared to the high CEC group (*p* ≤ 0.05). Further, we found that the low CEC group had increased TB and non-calcified coronary burden (*p* = 0.001) compared to the high CEC cohort (Table 1). Combined, the low CEC group had smaller lipoprotein particles, more inflammation and worsened vascular health.

### Low cholesterol efflux capacity modulated high-density lipoprotein and low-density lipoprotein subgroups

Since the low CEC group had a significant shift in concentration from large to small HDL and LDL, we assessed which HDL and LDL subgroups were modulated by poor CEC utilizing the advance LP4 deconvolution algorithm. We first determined the high CEC group had larger HDL and LDL particles according to size (*p* < 0.001) compared to the low CEC group. We found that there were significant increases in the concentration of large and medium HDL (*p* < 0.001) that was driven by increases in each subgroup (*p* ≤ 0.04). However, no change was observed in the small HDL group comparing low CEC to high CEC, concomitant with slight, non-significant, changes in the small subgroups.

The concentration of large LDL was higher in the high CEC group (*p* < 0.001). Additionally in the low CEC group, the concentration of medium LDL was lower (*p* = 0.001) and small LDL was elevated (*p* = 0.003) compared to the high CEC group. Within the small LDL subgroup, the 19.5 nm particle was increased in the low CEC group compared to the high CEC group (*p* = 0.009; Table 2).

**Table 2:** Comprehensive lipid panel using novel LP4 algorithm of psoriasis stratified by median CEC (0.95).

Lipoprotein size, nm	Diameter range (nm)	Low CEC, *n* = 110	High CEC, *n* = 103	*p*-value
HDL	7.5–13	20.7 ± 0.6	21.0 ± 0.6	<0.001***
LDL	19–23	8.8 (8.6–9.0)	9.0 (8.8–9.4)	<0.001***
**HDL Concentration, μmol/L**				
Total HDL	7.5–13	19.9 (18.5–22.2)	22.0 (19.9–25.1)	<0.001***
Large HDL	10.3–13	1.5 (1.0–2.2)	2.1 (1.3–4.1)	<0.001***
H7P	12.0	0.2 (0.1–0.5)	0.3 (0.1–0.7)	0.01*
H6P	10.8	0.2 (0.0–0.5)	0.4 (0.1–1.1)	<0.001***
H5P	10.3	0.8 (0.3–1.6)	1.2 (0.6–2.3)	0.02*
Medium HDL	8.7–9.5	2.8 (1.9–4.5)	4.3 (2.8–5.9)	<0.001***
H4P	9.5	1.5 (0.9–2.1)	2.1 (1.1–3.3)	0.002**
H3P	8.7	1.1 (0.0–2.5)	1.8 (0.3–3.5)	0.04*
Small HDL	7.4–7.8	15.1 (12.8–17.8)	15.4 (12.5–18.1)	0.99
H2P	7.8	11.1 (9.0–12.6)	11.7 (8.9–14.1)	0.09
H1P	7.4	3.9 (2.2–5.7)	3.2 (0.9–5.3)	0.06
**LDL concentrations, nmol/L**				
Total LDL	19–23	1421.0 (1179.0–1693.0)	1499.0 (1162.0–1792.0)	0.21
Large LDL	21.5–23	183.0 (74.0–398.0)	336.0 (192.0–587.0)	<0.001***
L-22.5	22.5	201.6 (73.8–410.8)	324.1 (163.4–491.5)	0.006**
Medium LDL	20.5–21.4	233.3 ± 215.3	349.1 ± 302.8	0.001**
M-21.0	21.0	185.8 (29.5–354.4)	226.5 (49.2–534.0)	0.12
Small LDL	19–20.5	878.0 (571.0–1252.0)	677.0 (413.0–979.0)	0.003**
S-20.5	20.5	450.0 ± 319.9	428.4 ± 341.9	0.64
S-20.0	20.0	0.0 (0.0–67.3)	0.0 (0.0–24.8)	0.24
S-19.5	19.5	0.0 (0.0–98.7)	0.0 (0.0–0.0)	0.009**
S-19.0a	19.0	24.7 (0.0–171.3)	27.8 (0.0–152.9)	0.42
S-19.0b	19.0	47.4 (0.0–166.1)	37.6 (0.0–212.1)	0.77

Values reported in the table as means ± SD or median (IQR). Low CEC defined as ≤ 0.95 (median CEC). ^*^p ≤ 0.05 deemed significant. ^**^p<0.01. ^***^p<0.001. LP4, LipoProfile 4; CEC, cholesterol efflux capacity; nm, nanometer; HDL, high-density lipoprotein; LDL, low-density lipoprotein; H1P-H7P, HDL subgroups by size; L, large; M, medium; S, small.

### High-density lipoprotein and low-density lipoprotein size associates with cholesterol efflux capacity, vascular inflammation and non-calcified burden

Next, we asked if lipoprotein size associated with HDL function, VI, and NCB in psoriasis (Table 3). In linear regression models, HDL size associated with CEC in unadjusted (*β* = 0.29, *p* < 0.001) and fully adjusted models (*β* = 0.23, *p* < 0.001). Moreover, HDL size had an inverse relationship with VI in unadjusted models (*β* = −0.29, *p* < 0.001) and fully adjusted models (*β* = −0.16, *p* = 0.03). Finally, HDL size had an inverse relationship with NCB in unadjusted models (*β* = −0.33, *p* < 0.001) and fully adjusted models (*β* = −0.19, *p* = 0.02). (Table 3).

**Table 3:** High density lipoprotein size regression analysis with CEC, VI, and NCB.

HDL size	*β*	*p*-value	LDL size	*β*	*p*-value
CEC	0.29	<0.001***	CEC	0.30	<0.001***
CEC + Model 1	0.22	0.001***	CEC + Model 1	0.28	<0.001***
CEC + Model 2	0.23	0.001***	CEC + Model 2	0.29	<0.001***
CEC + Model 3	0.23	0.001***	CEC + Model 3	0.29	<0.001***
VI	−0.29	<0.001***	VI	−0.29	<0.001***
VI + Model 1	−0.21	0.004**	VI + Model 1	−0.25	0.002**
VI + Model 2	−0.17	0.02*	VI + Model 2	−0.19	0.02*
VI + Model 3	−0.17	0.02*	VI + Model 3	−0.18	0.02*
NCB	−0.32	<0.001***	NCB	−0.35	<0.001***
NCB + Model 1	−0.21	0.006**	NCB + Model 1	−0.34	<0.001***
NCB + Model 2	−0.19	0.02*	NCB + Model 2	−0.32	<0.001***
NCB + Model 3	−0.19	0.02*	NCB + Model 3	−0.31	<0.001***

Model 1: Age, Sex, and Waist to hip Ratio. Model 2: Age, Sex, Waist to hip Ratio, CEC, hs-CRP. Model 3: Age, Sex, and Waist to hip Ratio, CEC, hs-CRP, Biologic Treatment and Statin Treatment. NCB presented as an average burden measured in the left anterior descending, left circumflex and right coronary arteries, and was log-transformed. *p ≤ 0.05, **p < 0.01, ***p < 0.001 deemed significant. HDL, high-density lipoprotein; LDL, low-density lipoprotein; CEC, cholesterol efflux capacity; VI, vascular inflammation; NCB, non-calcified coronary burden; Hs-CRP, C-reactive protein.

Similar associations were observed between LDL size and CEC, VI, and NCB (Table 3). LDL size associated with CEC in unadjusted models (*β* = 0.30, *p* < 0.001) and in fully adjusted models (*β* = 0.29, *p* < 0.001). Additionally, LDL size associated with VI in unadjusted models (*β* = −0.29, *p* < 0.001) and fully adjusted models (*β* = −0.18, *p* < 0.001). Similar to HDL size, LDL size associated with NCB in both unadjusted models (*β* = −0.35, *p* < 0.001) and in fully adjusted models (*β* = −0.31, *p* < 0.001; Table 3).

### High-density lipoprotein and low-density lipoprotein sizes are correlated

**Figure 1:**
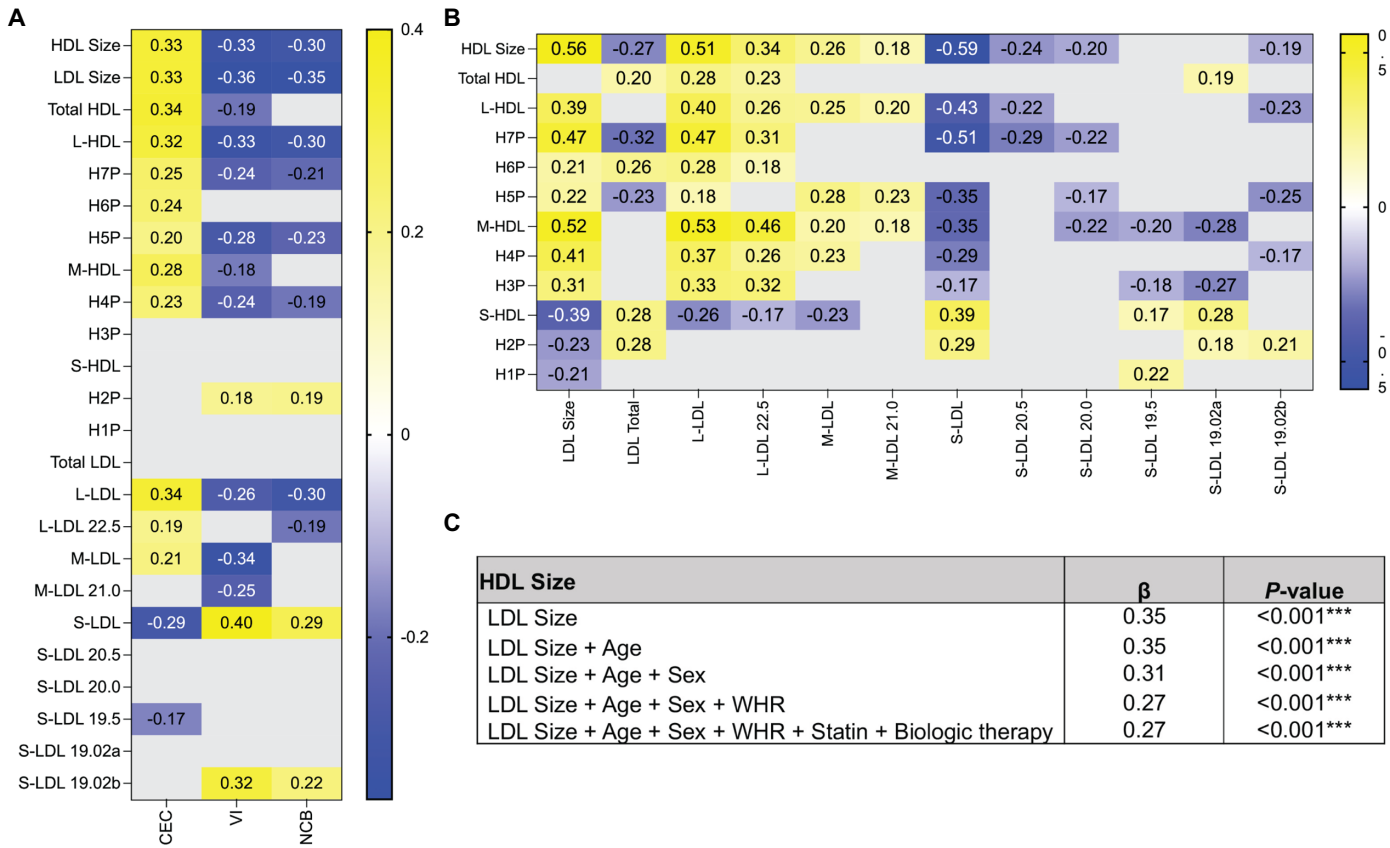
The relationship between VI and NCB and small and large lipoproteins differs in directionality. **(A)** Spearman correlation coefficients were determined between HDL and LDL subgroups and CEC, VI, and NCB. The correlation coefficients displayed represent significant associations. **(B)** Spearman correlation coefficients were calculated between the concentrations of HDL and LDL within subgroups to determine if the concentrations of each subgroup were interdependent. **(C)** Linear regression analysis of HDL and LDL size show that lipoprotein size has a robust relationship. All data in heatmap are expressed as Spearman’s correlation coefficient. Only significant values are reported. *p* ≤ 0.05 deemed significant. ***p < 0.001. HDL, high-density lipoprotein; LDL, low-density lipoprotein; H1P-H7P, HDL subgroups by size; L, large; M, medium; S, small; CEC, cholesterol efflux capacity; VI, vascular inflammation; NCB, non-calcified coronary burden; WHR, Waist-to-Hip ratio.

Significant shifts were observed in the concentrations of HDL and LDL subgroups. Therefore, we asked if the HDL and LDL subgroups were associated with CEC, VI, and NCB. We observed strong correlations between large HDL and large LDL and CEC, and inverse relationships with VI and NCB (all displayed *p* ≤ 0.05; Figure 1A). Moreover, we observed a positive correlation between the small H2P (7.8 nm) HDL, small LDL and VI and NCB (Figure 1A). Furthermore, we observed stepwise decreases in the strength of the relationship between HDL particle size and VI and NCB, that changed directionality with smaller HDL particles (all displayed *p* ≤ 0.05; Figure 1A). We found that the size of HDL, LDL and their subgroups are highly dependent, with the concentration of smaller HDL correlating to the concentration of smaller LDL and larger HDL to larger LDL (all displayed *p* ≤ 0.05; Figure 1B). Finally, in unadjusted (*β* = 0.35, *p* < 0.001) and fully adjusted regression models (*β* = 0.27, *p* < 0.001) HDL size was associated with LDL size (Figure 1C).

## Discussion

Utilizing a prospective study design, we demonstrate four major findings: (1) patients with lower CEC have an altered lipoprotein profile, higher inflammatory markers, and metabolic dysregulation; (2) this corresponds to a significant shift from larger to smaller lipoprotein particles; (3) HDL and LDL size are associated with CEC, VI, and NCB; (4) HDL and LDL size have an interdependent relationship. Combined, these findings provide unique insight into the importance of lipoprotein function and size in CV risk and suggest the distribution of HDL and LDL size should be considered when developing therapeutic strategies to improve CV outcomes in psoriasis.

In this study, we determined that psoriasis patients with poor cholesterol transport had more systemic inflammation and worse vascular health by VI and NCB. Preclinical models of atherosclerosis have demonstrated an interdependency between cholesterol trafficking and atherosclerosis suggesting insufficient cholesterol trafficking may drive inflammatory atherogenesis (13, 14). Further, the Canakinumab Anti-inflammatory Thrombosis Outcome Study (CANTOS) showed that in patients with a previous myocardial infarction concomitant with systemic inflammation, IL-1β inhibition reduced coronary disease (37). IL-1β is produced upon NOD-, LRR- and pyrin domain-containing protein 3 (NLRP3) inflammasome activation which is regulated by cholesterol efflux pathways (14). Moreover, poor cholesterol transport upregulates myelopoiesis in the bone marrow, which is consistent with our observation that in psoriasis, low CEC is associated with more neutrophils and elevated bone marrow activity (13). These findings support an intersection between lipid transport and inflammation in a clinical model and warrant future studies in understanding the mechanistic role of cholesterol transport in early onset atherosclerosis often observed in psoriasis.

Poor cholesterol transport associated with a significant shift from large to small lipoproteins. Moreover, the size of HDL and LDL had relationships with CEC, VI, and NCB. Interestingly, large HDL and LDL were inversely associated with VI and NCB and small HDL and LDL had a positive correlation. These findings are consistent with numerous studies including the Multiple Risk Factor Intervention Trial (MRFIT) that evaluated the relationship of metabolic syndrome and coronary artery disease mortality (38). The MRFIT study reported that higher levels of medium HDL specifically were associated with a lower risk of death (38). In the Malmo Diet and Cancer Study Cardiovascular Cohort, a prospective community-based epidemiological cohort, three components of CVD risk that is an atherogenic lipoprotein phenotype; decreased large HDL, increased small LDL and increased triglycerides were reported (39). Another study showed that the concentration of small LDL is more accurate than total LDL levels at determining coronary artery disease in both males and females (40). In the Framingham Heart Study, a sex-specific cross-sectional examining the relationship between small LDL and metabolic syndrome showed that in males and females, small LDL increased with increasing metabolic syndrome traits, which associated with CVD event rate (41). Further, methods in machine learning reported that the larger LDL particle may be protective, while the smaller LDL particles were not only predictive of coronary artery disease, but the smaller the particle, the higher the odds ratio for coronary artery disease development (42). These findings are consistent with the low CEC group in psoriasis that have an increased prevalence of metabolic syndrome, and smaller HDL and LDL lipoproteins that associates with increased VI and NCB.

Our study had certain strengths and limitations. We did not have hard cardiovascular events as our outcome. However, a major strength of our study is the ability to phenotype and quantify both inflammation in the aorta and coronary artery burden using non-invasive techniques and assess their relationship with lipoprotein function and size. FDG PET/CT and CCTA offer robust non-invasive imaging methods to characterize coronary artery disease and plaque composition (43). Patients with incident acute coronary syndromes have been shown to have higher arterial FDG uptake as well as higher total coronary plaque volume by CCTA, predominantly composed of rupture prone non-calcified plaques (44–46). Furthermore, psoriasis patients have been shown to have increased coronary plaque burden by CCTA when compared to 10-year older hyperlipidemic patients and increased arterial FDG uptake which associates with this heighted plaque (25, 47). Pathophysiologic pathways for increased coronary artery disease in psoriasis patients include impaired HDL-CEC (19). Furthermore, CEC is a reliable marker of HDL function (48) and improves atherosclerosis risk prediction beyond coronary artery calcium score, family history of coronary artery disease, and high sensitivity C-reactive protein (49).

In conclusion, we demonstrated that in psoriasis patients at high risk of prospective cardiovascular events, low CEC is associated with smaller HDL and LDL subgroups. Further, HDL and LDL size is associated with markers of subclinical atherosclerosis and are interdependent. These findings highlight the role of lipoprotein size and NMR derived subfractions as reliable biomarkers to improve our understanding of how HDL and LDL function and size relates to subclinical atherosclerosis, and for the prevention of future atherosclerotic cardiovascular disease risk.

## Data availability statement

Upon request the raw data supporting the conclusions of this article will be made available by the authors, without undue reservation.

## Ethics statement

The studies involving human participants were reviewed and approved by National Institute of Health. The patients/participants provided their written informed consent to participate in this study.

## Author contributions

HT and NM: conceptualization, project administration, supervision, validation. HT, RP, and AB: data curation. NM: formal analysis and funding acquisition. HT, RP, AB, and NM: investigation, software, and writing – original draft preparation. HT: methodology. HT, MP, and NM: resources. HT, RP, and NM: visualization. HT, RP, AB, AG-C, MP, and NM: writing – review and editing. All authors contributed to the article and approved the submitted version.

## Funding

This study was funded by the National Heart, Lung and Blood Institute Intramural Research Program in Bethesda, Maryland (HL006193-07).

## Conflict of interest

NM has served as a consultant for Amgen, Eli Lilly, and Leo Pharma receiving grants/other payments; as a principal investigator and/or investigator for AbbVie, Celgene, Janssen Pharmaceuticals, Inc., and Novartis receiving grants and/or research funding. AG-C has served as a consultant for Abbie, Janssen, Novartis, Almirall, Celgene and Leo Pharma receiving grants/other payments.

TThe remaining authors declare that the research was conducted in the absence of any commercial or financial relationships that could be construed as a potential conflict of interest.

## Publisher’s note

All claims expressed in this article are solely those of the authors and do not necessarily represent those of their affiliated organizations, or those of the publisher, the editors and the reviewers. Any product that may be evaluated in this article, or claim that may be made by its manufacturer, is not guaranteed or endorsed by the publisher.

## Abbreviations

CEC: Cholesterol efflux capacity
CVD: Cardiovascular disease
VI: Vascular inflammation
NCB: Non-calcified coronary burden
HDL: High-density lipoprotein
LDL: Low-density lipoprotein
nm: Nanometers
CETP: Cholesteryl ester transfer protein
Apo-A1: Apolipoprotein A-1
NMR: Nuclear magnetic resonance
FDG PET/CT: 18F-fluorodeoxyglucose positron emission tomography computed tomography
CCTA: Coronary computed tomography angiography
PACI: Psoriasis Atherosclerosis and Cardiometabolic Disease Initiative
SUVmax: Standardized uptake value
TB: Total burden
LP4: LipoProfile 4
HOMA-IR: Homeostatic model for insulin resistance
IL6: Interleukin-6
hs-CRP: High-sensitivity C-reactive protein
TNF-α: Tumor necrosis factor-alpha
NLRP3: NOD-, LRR- and pyrin domain-containing protein 3
